# Does the Faculty’s Perception of Gender Discrimination Relate to Its Assessment of Organizational Democracy in the University?

**DOI:** 10.3390/bs13060450

**Published:** 2023-05-30

**Authors:** Elizabeth Troncoso, Wendolin Suárez-Amaya, María Ormazábal, Luis Sandoval

**Affiliations:** 1 Department of Chemistry, Universidad Tecnológica Metropolitana, Las Palmeras 3360, Ñuñoa, Santiago 7800003, Chile; 2 Programa Institucional de Fomento a la Investigación, Desarrollo e Innovación, Universidad Tecnológica Metropolitana, Ignacio Valdivieso 2409, San Joaquín, Santiago 8940577, Chile; 3 Department of Organizational Management, Universidad Tecnológica Metropolitana, Dr. Hernán Alessandri 722, Providencia, Santiago 7500998, Chile; wsuarez@utem.cl; 4 Gender and Equity Program, Universidad Tecnológica Metropolitana, Avda. Dieciocho 145, Santiago 8330300, Chile; mormazabal@utem.cl; 5 Office of the Vice President of Academic Affairs, Universidad Tecnológica Metropolitana, San Ignacio de Loyola 160, Santiago 8330366, Chile; lsandoval@utem.cl

**Keywords:** organizational behavior, university faculty, gender, organizational democracy, gender discrimination

## Abstract

This work aimed to study the relationship between the perception of organizational democracy and gender discrimination at a Chilean public university. It is known that organizational democracy is not only about organizational life but also about democratic perceptions, attitudes, and behaviors in social life, as found in academic contexts. The methodology used factor analysis and descriptive and inferential statistical techniques to analyze data from a survey administered to 704 university faculty members, with a response rate of 58.1%. The gender distribution of this respondent population was 67% male and 37% female, values equivalent to the Chilean public university system (60% and 40%, respectively). The results highlight the importance of gender perspective in higher education. Indeed, academics who perceive greater gender discrimination toward women appreciate the deployment of organizational democracy to a lesser extent. Moreover, a high perception of discrimination on the part of women is confirmed (46%), them being, in turn, the ones who show a greater predisposition toward gender equality. This research intends to contribute to the development of strategies to remove obstacles to gender equality and improve the commitment of the academic community to institutional progress.

## 1. Introduction

The establishment of the gender perspective as part of the dynamics of institutions is related to the human rights approach. This approach plays an ambivalent role, which, on the one hand, is a theoretical–conceptual reference for its application in particular contexts and, on the other, is an expression of social relations, cultural interconnection, and ethical agreements on equity/equality. In this sense, environments that allow the exercise of these fundamental freedoms promote changes in all spheres of action and spaces for the deepening of democracy [1,2,3].

Chile has experienced important economic, social, cultural, and demographic transformations in the last decades. For example, poverty levels decreased by 17.2 percentage points between 2009 and 2022, reaching 10.2% today [4]. Access to higher education has also become more widespread due to public policies and support mechanisms for educational, labor, and political inclusion for the most disadvantaged socioeconomic sectors, such as indigenous peoples, who represent 12.8% of the Chilean population [5].

Economically, there is a growing integration of women into the labor market, close to 41.8% in 2021 [6], contributing to their economic autonomy and addressing inequality and poverty [7,8]. However, wage gaps persist, as they receive incomes 21.7% lower than men [6], as well as gaps in participation in decision-making positions [9].

In sociodemographic terms, 51.05% of the Chilean population comprises women [10]. The reduction in the number of children per woman and the increase in life expectancy place the country in an advanced stage of demographic transition characterized by a gradual aging of the population, and those over the age of 60 are projected to comprise 31.2% of the population by 2050 [11]. It is also important to note that Chile is a country in Latin America with the second-highest proportion of migrants [12].

Regarding gender, Chile adheres to international conventions and treaties on gender equality, such as the Universal Declaration of Human Rights [13], the Declaration on the Elimination of Violence against Women [14], and the Beijing Declaration [15], among others. It has also made progress in legal provisions such as Law No. 21.369 of 2021 [16], which regulates sexual harassment, violence, and gender discrimination in higher education.

The incorporation of the gender perspective in universities implies significant structural changes if we want to move toward more open and democratic organizations. In such a context, the perceptions of the academic community acquire a strategic character because they reflect the level of resistance to adapting to new cultural patterns. Various investigations show that the perception of the organization impacts key aspects of management, such as attitudes and behaviors [17], psychological capital [18], job satisfaction [19], meaningful work [20], and knowledge sharing [21]. According to Adobor (2020) [22], institutions that promote inclusion, transparency, and shared decision making as part of their structure and culture of organizational democracy show greater openness to include internal and external interest groups in their strategic definitions.

In this order of things, it could be assumed that an organization that is perceived as undemocratic or with a low level of perception of organizational democracy (POD) risks weakening the commitment of its employees, with the consequent loss of institutional effectiveness. At the university level, faced with a low POD, academics could perceive that institutional problems are addressed without considering the community’s interests. An inherent factor in organizational democracy is the absence of gender discrimination, being of interest to verify the relationship between the perception of gender discrimination (PD) of the academic community and the POD. In the same sense, it is worth asking if there is a relationship between the POD and the attitude toward gender roles (ATGR) exhibited by its academics.

Attempting to further understand the above-mentioned interactions, the objective of this study was to determine the relationship between the perception of organizational democracy, the perception of gender discrimination, and the attitudes toward gender roles for the faculty of a public university in Chile, controlling for a personal and social variable vector in the institution and disciplinary field (STEM and no-STEM) of the faculty in this perception, to contribute to the development of strategies for removing obstacles to gender equality.

### 1.1. Theoretical Background

#### 1.1.1. Discrimination

Discriminatory behavior is understood as biased treatment based on characteristics such as race, color, ethnic origin, age, gender, etc. [23], also including the different forms of sexual harassment, sexual assault, and sexism in general [24]. These experiences have been negatively associated with job performance (job satisfaction, commitment, and organizational outcomes), psychological health (well-being and distress), and physical health (health satisfaction and physical symptoms) [25]. Discrimination is also observed in organizations open to people of diverse sexes/genders, who experience differential treatment that induces a continuous devaluation and marginalization of the person [26,27].

Sexual harassment and sexism are accentuated in male-dominated organizations and professions. This is often the case in universities [28,29,30,31,32,33], despite demonstrating that university teaching staff have a favorable attitude toward the culture of equality. This has been observed to be particularly true for female teaching staff [34,35,36]. Sexual harassment is a serious problem in higher education institutions, a situation heightened by its hierarchical organization, precarious working conditions, normalization of gender violence, toxic masculinity, culture of silence, and lack of active leadership [37].

Most of the research regarding gender discrimination has been conducted in North America, confirming a lack of knowledge regarding these topics in other latitudes such as South America and Asia. This prevents having a more global representation of the problem to design and implement focused interventions [38]. This occurs despite knowing that its effects impact the organizational environment and have a systemic nature; that is, they imply costs for higher education institutions due to a greater turnover of their employees, a deteriorated perception of positive changes in the university, and less participation [37,39].

#### 1.1.2. Organizational Democracy

Organizational democracy (OD) is a managerial approach that shares power with community representatives to achieve an organizational climate based on transparency and tolerance. It has a few variants for analysis, one of them being participation in decision making and management. Another is the relationship between organizations’ economic and social aspects, affecting democratic practices in social life. Thus, organizational democracy is not only about organizational life but also about democratic perceptions, attitudes, and behaviors in social life [19,20,21,40,41,42].

In the academic field, the research developed by Çopur and Atanur Baskan (2020) [43] found a high, negative, and statistically significant relationship between academics’ perception of organizational democracy and organizational cynicism, the latter being understood as a negative attitude of the employee toward the employing organization based on the belief that the organization lacks integrity. For their part, Turabik and Atanur Baskan (2020) [44] stated that a university will perform effectively in the education, research, and social service it develops if these basic duties are performed in an environment where academic and administrative staff can work calmly and students can continue their learning activities in a favorable climate, all of which is directly related to the level of organizational democracy.

Organizational democracy indicators can be conceptualized and measured in three ways: (i) formal participation of employees in organizational decisions, (ii) participation of employees in collective ownership, and (iii) perception of employees regarding their participation in the organization’s decision making [20]. This last indicator is one of special interest in our study.

#### 1.1.3. Gender and Gender Roles

Gender equality refers to “the equal rights, responsibilities and opportunities of women and men and girls and boys. It implies that the interests, needs and priorities of both women and men are considered, recognizing the diversity of different groups of women and men” [45]. Guaranteeing gender equality in higher education institutions requires a community that is sensitive and aware of the issue [46]. The above inevitably implies a cultural change in the relational practices of organizations, particularly considering that universities are mostly male institutions with a patriarchal exercise of power [47] and that they have operated with stereotypes in the gender roles traditionally assigned to the sexes, with its consequent effects on the naturalization of discrimination, inequities, and violence [48,49,50,51].

Some obstacles to gender equality identified in the academic space are the diversity of perceptions regarding gender equality [52,53], the scarce formal preparation for the implementation of the gender perspective in university teaching, the lack of consensus regarding gender mainstreaming in institutions [54], and ignorance of the problem [55]. Added to this is the predominance of gender stereotypes, which represents a cultural bias that is difficult to eradicate [56,57,58,59]. As McCarry and Jones (2022) [60] point out, cultures and practices of gender inequality have been altered but not dismantled. The problem raised is accentuated when considering the perceptions of women academics within their institutions, which include an unwelcoming and threatening academic climate, and the scarce or null existence of support networks for the professional success of women [61,62,63,64].

The traditional role of women in the domestic space has been identified as the main and hidden cause of their exclusion in political, educational, and artistic spaces, among others [65]. Various symbolic and social mechanisms perpetuate the sexual division of labor, making it seem necessary and immovable, which is known as the gender regime [48]. Therefore, identifying what happens with the perception, attitudes, beliefs, and ideas regarding gender roles (ATGR) is a priority to initiate actions toward more egalitarian relationships that respond to each organization’s particular needs. ATGR are referred to as “beliefs regarding the appropriate roles for men and women”. Gender beliefs are important because they are one piece of the narrative of increasing gender equality in society [66,67]. Gender roles are built in relation to others and in a specific context, later becoming naturalized in the daily practices of an organization [49]. A traditional/conservative perspective on gender roles, based on gender bias and stereotypes, could imply that universities do not promote effective politics to foster their communities’ intellectual development and open-minded thinking, revealing the need for more sensitivity to the equality of gender roles.

Based on the above, and to the best knowledge of the authors, it can be concluded that there is little research that establishes a relationship between organizational democracy and gender variables in the university environment, this being precisely the contribution offered by this research.

## 2. Materials and Methods

### 2.1. Data

The data for this study come from a survey administered online in May 2021 to the faculty members of a public university in Chile, collecting information about gender relations and gender equity conditions. This public university is situated in the Metropolitan Region of Chile, serving students from across the country. Its student enrollment is around 10,000, and it strongly emphasizes STEM education.

The survey comprised 42 questions, which were grouped into the following categories: (1) general characterization, (2) workplace environment from a gender perspective, (3) procedures related to situations of gender discrimination, and (4) perception regarding gender equity. For constructing the gender relations questionnaire, surveys used in the diagnoses of gender relations conducted by other Chilean universities were used as a basis, along with questions included in national surveys on gender equality. University officials authorized the survey application, which incorporated informed consent, the purpose of the study, anonymity in the data collection process, and the voluntariness of participation. The population corresponds to the university faculty in 2021, composed of 704 academics. A total of 409 valid responses were received, representing a response rate of 58.1% of the total population. Since the sample has non-probability of voluntary responses, coverage and post-stratification corrections were made to better represent the population and reduce possible bias [68].

All subjects gave their informed consent for inclusion before they participated in the study. The study was conducted in accordance with the Declaration of Helsinki, and the protocol was approved by the Ethics Committee of Universidad Tecnológica Metropolitana (protocol code CEC-23-10).

### 2.2. Data Analysis

The data were analyzed using descriptive and inferential statistical techniques and the ordinary least squares (OLS) method for multiple regression. Factor analysis was applied to define the scales used in the study: (i) perception of organizational democracy (POD), (ii) perception of discrimination against heterosexual men (PDHM), (iii) perception of discrimination against heterosexual women (PDHW), (iv) perception of discrimination against sexual and gender minorities such as lesbian, gay, bisexual, transgender, and queer (LGBTQ) (PDLGBTQ), and (v) attitudes toward to gender roles (ATGR). In this study, the last one is focused on the cultural narrative, which is what people believe [66,67].

The outcome of interest is the university faculty’s POD. The dependent variables corresponded to the PDHW, PDHM, PDLGBTQ, and ATGR indexes. Control variables included gender, age, employment seniority in the university, disciplinary field, belonging to indigenous peoples, disability condition, nationality, and parenthood. In the case of the gender control variable, only the categories “male” and “female” were considered, which represented >99% of individuals who were invited to participate. The gender distribution of the population is equivalent to the Chilean public university system. According to the diagnosis of trends in gender gaps in Chile’s public universities [69], 60% of the faculty comprises men, while 40% are women. The survey items used to construct each index are shown in Table 1, and the sample characteristics are described in Table 2.

The missing values were imputed with the average value of the valid responses in the indexes when these were equal to or greater than 60% of the responses. Otherwise, they were not considered in the calculation of the average value of each record. This approach assumes that when a person skips an item, the individual’s own average score for the answered items is a reasonable substitute value for that item.

The construction of the indexes considered the quantification of the responses in a 4-option Likert-type format, assigning them scores from 1 to 4. Factor analysis was applied to choose the component items of each index. Then, the average score, standard deviation, and associated difference for each index were calculated. A cut-off of 2.5 was elected to facilitate the analysis, corresponding to the scores’ intermediate value. It divides the respondents’ positions into those above or below this cut-off score, revealing favorable or unfavorable perceptions about the study’s variables.

Calculations of statistical significance and association estimation between variables were made based on considering enough similarity in the distribution of control variables in the sample and the population because it is a non-probability sampling [68].

## 3. Results

### 3.1. Distribution of ATGR, POD, and PD by Gender and Disciplinary Field

The distribution of ATGR, POD, and PD according to gender and disciplinary field is shown in Table 3. ATGR exhibited significant differences between men and women, the latter being more open to gender role equality (3.47 women vs. 3.37 men). A higher ATGR index score implies higher affinity with the equality of gender roles. ATGR also revealed differences by the disciplinary field, being significantly lower among STEM (3.34) than non-STEM faculty (ATGR = 3.46). Notwithstanding, considering a cut-off of 2.5, the majority of the university faculty have a favorable view of the ATGR (~99%) independent of gender and discipline.

The mean POD shows a significant difference between women and men, with values of 2.29 and 2.44, respectively (Table 3). Male faculty members are equally distributed among those who perceive a high or low POD, while women are more likely (61%) to have a low POD. In addition, POD was significantly higher in the STEM (2.48) than in the non-STEM group (POD = 2.32). Both cases had average values below the cut-off of 2.5; however, the first one is equally divided between high and low POD, while the other group mainly (~58%) perceives low organizational democracy. These findings imply that a large group of university faculty, especially women, perceive the university as a rigid organization, not very open to participation in decisions or actions that favor institutional work.

The PD analysis was performed concerning the perception of women and men toward three subgroups: heterosexual men, heterosexual women, and gender diversities. For this index, the lower the score, the greater the perception of discrimination. The discrimination perceptions toward heterosexual men (PDHM), heterosexual women (PDHW), and sexual and gender minorities (PDLGBTQ) exhibit statistically significant differences when analyzed by gender (women vs. men), as shown in Table 3. Men perceive less discrimination than women toward heterosexual women and the LGBTQ group, behavior contrary to men’s perceptions of themselves. Regarding the discipline, only PDHW and PDLGBTQ show significant differences by disciplinary field, whereas the non-STEM professors perceive more discrimination against women and the LGBTQ collective. It should be noted that the mean value of PDHM is the highest (~3.4) among the three groups (Table 3). This value is almost 1 point above the cut-off of 2.5, denoting a perception of low discrimination toward heterosexual men. The percentage distribution around the cut-off reveals that only 12.4% of male members perceive themselves as discriminated against. This percentage drops even further from the perception of women, where only ~3.5% think that heterosexual men suffer discrimination. A similar situation occurred in the distributions by disciplinary field, where only about 9% of teachers believe that male teachers are discriminated against. A different situation occurs regarding the perception of discrimination toward heterosexual women (PDHW), where both men and women have a higher perception of discrimination, with values of 2.97 and 2.54, respectively, which are notably lower in women than in the case of men. When the percentage distribution around the cut-off is analyzed, almost 46% of women perceive high discrimination toward them. In contrast, only 22% of men agree with this position. The distribution by discipline demonstrates that the non-STEM faculty perceive more significant discrimination (~35%) toward women than ~22% of STEM professionals.

Regarding the perception of discrimination against sexual and gender minorities (PDLGBTQ), there is also high perceived discrimination against this group, with values of 2.59 in women and 2.89 in men. In this sense, women are more categorical (~36%), although they think they are more discriminated against. On the other hand, men believe that sexual diversities are more discriminated against than women (~25% vs. ~22%). Further, university faculty in non-STEM disciplines manifested a greater perception of discrimination toward the LGBTQ group than those in the STEM fields (32% vs. ~23%).

### 3.2. Distribution of ATGR, POD, and PD by Age and Employment Seniority

The ATGR, POD, PDHM, PDHW, and PDLGBTQ indexes were correlated with age and employment seniority (Table 4). ATGR shows a significant and inverse correlation with age, i.e., the younger faculty members (<40 years) are proportionally more supportive of equality of gender roles than their older peers (≥40 years). This relationship was not observed between ATGR and employment seniority at the university, even though there is a significant difference between the averages of the middle and upper groups. In the case of POD, there is no significant correlation with age (*p* > 0.05). In contrast, an opposite result was found for employment seniority, which was negative, with significant differences between the mean POD values of the “low-middle” and “low-high” segments. In short, faculty members who have been at the university longer (>5 years) have a lower perception of organizational democracy (mean POD~2.3), placing them below the cut-off.

Regarding PDHM, no relationship exists between this index and age or employment seniority (Table 4), with a uniform consensus among university faculty that this is minimal (mean 3.3 out of 4.0). In contrast, perceived discrimination toward women is higher (mean 2.8), especially among young academics (2.6), with significant differences between the latter and their older peers. In turn, PDHW did not exhibit a significant correlation with employment seniority. On the other hand, PDLGBTQ has a positive relationship with the age and employment seniority of the faculty. As explained earlier, a higher discrimination index score implies a lower perception of discrimination. In this case, faculty members of higher age and greater employment seniority perceive less discrimination toward sex/gender-diverse individuals.

### 3.3. Regression Model for Determining the Impact of Different Indexes and Control Variables on POD

Finally, and to better understand the relationship between the variables analyzed, a linear regression model was fitted considering POD as the outcome and ATGR, PDHM, PDHW, and PDLGBTQ as dependent variables, together with the control variables of gender, age, employment seniority, disciplinary field, indigenous people, disability condition, nationality, and parenthood (Table 5). These control variables were considered because of their potential confounding effect on the relationship between POD and perceived discrimination.

A model including PDHW, PDLGBTQ, STEM area, employment seniority, belonging to indigenous peoples, and disability condition explains 22% of the variance of POD with F (12, 279) = 11.25, *p* < 0.001. The ATGR, PDHM, gender (female), age, nationality, and parenthood variables do not have a statistically significant effect. PDLGBTQ (β = 0.091) and employment seniority (β = −0.0071) have a weak but statistically significant effect on POD. The strongest predictors were PDHW (β = 0.2174, *p* < 0.001), disability condition (β = −0.5192, *p* < 0.01), and disciplinary field (STEM) (β = 0.1031, *p* > 0.01).

It can be concluded that the higher the perception of discrimination against women and people outside the gender binary (higher scores on the respective indexes), the lower the evaluation of POD. In short, people who perceive less discrimination toward these groups show a higher value of organizational democracy at the university. It is also worth highlighting that the STEM faculty have a higher assessment of organizational democracy. An opposite situation is observed when evaluating the impact of employment seniority on POD, although with a smaller effect size. The findings concerning people with disabilities demonstrate they tend to value organizational democracy less, which could reflect their dissatisfaction with politics or strategies at the university to include this group. This fact raises questions about improving institutional support to promote more inclusive workplaces. Finally, belonging to indigenous peoples implies a greater positive perception of organizational democracy with a significance of ~10%.

## 4. Discussion

Even with the limitations of the sample in terms of strict representativeness, the findings of the perception of organizational democracy are framed in what was stated by Eslen-Ziya and Yildirim (2022) [70], who conclude that the perception of the gender situation is related to the vision that one has regarding how hierarchical the institution is. The more hierarchical, the less democratic. The above is supported by the fact that the perception of gender equality is directly interrelated with the sociocultural context [71,72], and, therefore, perceiving an unwelcoming and threatening academic climate, together with little or no existence of support networks, is a barrier to the professional development of women [61,62,63]. This is consistent with our findings that women perceive less organizational democracy than men. In general, the literature related to organizational democracy recognizes the impact of gender on management practice to make the most of the contribution of women to organizations or simply to recognize that difference as part of diversity [73].

The low POD of academics, regardless of their disciplinary field, could reflect, according to Turabik and Atanur Baskan (2020) [44], the need to implement practices that promote awareness of their rights and responsibilities as citizens, the acquisition of critical perspectives, and respect for diversity as a member of a globalized world. These are fundamental characteristics of a democratic way of life that universities must cultivate for their projection to society. The foregoing is key to favorably impacting the effectiveness of the institution, understood as the degree of fulfillment of its objectives to carry out its mission and function, and its efficiency, understood as the level of satisfaction of the interested parties and the good use of their resources [74].

The fact that academics in STEM areas perceive greater organizational democracy is consistent with the existence of preferences in the different disciplines regarding academic leadership for management in higher education institutions. It is well known that the discipline is a source of identity in the professional life of teachers and can produce variations in academic direction and management priorities [75], as well as in their social and epistemological orientations and political attitudes [76]. Disciplines such as sociology have been associated with emancipation rather than increased efficiency and tough management, and its practitioners are critical of effective, results-oriented management since this partly creates inequality or may go against democracy and equal opportunities in decision making. This contrasts with what happens in tougher disciplines (e.g., physics) that value pragmatic, direct, and even technical university management and where said management will be good as long as it works and does not affect personal values and interests or departmental traditions [77].

On the other hand, the lower organizational democracy perceived by academics with greater seniority in the institution could be related to the deterioration of their perception of quality in their workplace [78] or to the existing pressure from younger academics to abandon the seniority principle and move toward academic ranking systems based on the merit principle [79]. Older professors with higher academic ranks have proven to be more resistant to change, and professors with less seniority in higher education institutions (<10 years) are more prone to decision-making deprivation than their senior peers (>20 years), which impacts organizational effectiveness [80]. In any case, it has been recognized that organizations that differ in terms of seniority and age of their workers produce processes of sub-grouping by age that favor the perception of a negative climate of age discrimination, which is negatively related to the performance of the organization. This leads to some labor decisions being made based on the age of the workers [81], which can impact perceived democracy. However, two facts cannot be dismissed: (i) there are other pressures driven by age-related changes in work or social contexts (e.g., stereotypes or loss of status) [81], and (ii) in the context of higher education institutions, there is administrative pressure on the older group to retire, preferring the hiring of younger professors, who carry a lower cost due to their lower hierarchical rank but who could better adhere to certain institutional challenges [82]. These situations undoubtedly call for an improvement in practices in the management of organizational human resources to positively impact the efficiency and effectiveness of universities.

Regarding the perception of discrimination, our findings are in line with the results of other studies, especially when considering environments with a male predominance, as occurs in academic institutions [37]. Studies focused on the Chilean public university system reveal that gender discrimination is perceived by 64.5% of female academics, an opinion held by 35.5% of male academics [69], which reflects a similar context to the one revealed in this work (approximately 62% female and 38% male). Other studies outside the Chilean context have also shown that men tend to perceive situations of institutional or structural discrimination toward women to a lesser degree compared to the perception of women themselves [83,84,85]. A recent study concluded that among STEM scholars within the same institutional context, men differed in the ways they understood and acted toward gender issues [86]. This suggests that promoting gender diversity does not necessarily raise sensitivity toward gender equality among all men. For example, Carr et al. (2020) [87] found that female faculty members were more than 2.5 times more likely than male faculty members to perceive gender-based discrimination in the academic environment. These results are similar to those reported by Hill and Hurley (2022) [88].

For their part, the perception of men regarding discrimination toward sexual diversities is greater than the perceived discrimination toward women. Similar results have been reported by Gururaj et al. (2022) [89], one of the few studies that address the issue, which found that male teachers have a more favorable attitude toward the LGBT community than female teachers. Perhaps this attitude is due to the treatment given by the media, highlighting situations of tolerance toward sexual diversities. This effect has recently been addressed by Taracuk and Koch (2023) [90], who established that barriers to equality and inclusion for transgender and gender-diverse people can include a variety of factors, most notably discrimination in educational settings and labor.

Regarding the perception of discrimination toward men, be it their self-perception or that of women, it is found that studies on this topic are almost non-existent. In the work of Funk and Parker (2018) [91], although not focused on the self-perception of discrimination of men, the values reported agree with those identified in this study. The issue is also addressed in Korea by Lee et al. (2022) [92], who found men’s perception of discrimination toward themselves to be higher than that of women, which was associated with the specific cultural context of the country.

The results related to the perception of discrimination of academics in STEM areas were lower than those in non-STEM areas, which contradicts findings reported by Funk and Parker (2018) [91] and García-González et al. (2019) [93]. The explanation behind our results lies in the fact that aggregated data are presented in which women are underrepresented. When STEM women are isolated, they have a greater perception of discrimination.

In relation to the age of the teaching staff and the perception of discrimination, a consensus was found that men suffer less discrimination than women and the LGBTQ group regardless of age, which is consistent with previous studies [28,93]. Regarding variations with age, our findings establish that young academics have a greater perception of discrimination toward women than their older counterparts. This is in line with previous evidence indicating that younger teachers are more sensitive to gender issues, since from an early age, the issues of women’s rights and gender discrimination were a matter of debate, valuing more equal and diverse treatment regarding gender [88,94]. Similar evidence was reported by Kehn and Ruthig (2013) [95], although their findings were not set in an academic context.

Employment seniority is not related to the perception of discrimination toward women or men, which is contradictory to the results of Mihajlović Trbovc et al. (2022) [96], who find that newly admitted academics have high visions of inequality. In our results, the senior faculty reported a lower perception of discrimination toward sexual minorities. More research is required regarding these findings.

On the other hand, the results on ATGR are consistent with those of studies developed in various contexts [34,35,36,51], registering a high disposition to gender equality among academics, with this attitude being stronger in women because they are the ones who experience the most discrimination from having to overcome a series of obstacles of a political, religious, cultural, and social nature [97,98,99]. Likewise, ATGR is significantly higher in women and non-STEM academics, an issue that, as already mentioned, is related to the fact that women experience greater discrimination in work contexts and also have a sensitivity typical of those who belong to non-STEM areas whose field does not present the gender biases observed in STEM areas. Women in STEM fields face a constant need to prove their abilities to their peers, managers, and students [100,101]. Moreover, studies have concluded that, in terms of publications, the improvement is slower in those disciplines with a greater gender bias [102].

On the other hand, ATGR was significantly higher in younger scholars. These results coincide with other studies [88,94], whose explanatory hypotheses are linked to the fact that this age group has played its role under legislative initiatives for gender equality unlike their older peers less exposed to such practices. In addition, regardless of gender and disciplinary field, a highly favorable view of equality of gender roles was found (~99%). While this can be considered politically correct, it is difficult to pin down what causes cultural change, highlighting the need to build global indicators of cultural change in higher education institutions to assess progress toward gender equality [47]. However, the implementation of institutional gender policies, affirmative actions to correct inequalities, regulations stemming from laws and public policies aimed at gender equality, and efforts to increase knowledge on the matter are mechanisms that contribute to consolidating cultural change [103].

Finally, the linear regression model used to adjust the relationship between the variables of interest, controlling for possible confounders, confirms the previously discussed results. Although the model only explains 22% of the variance of the POD, it is significant and is acceptable when contrasted with other findings. Indeed, the variables that reflect a high rate of discrimination are more significant in the model (PDHW, disability status, and PDLGBTQ). The STEM disciplinary field variable also appears to be strongly significant, reflecting the more favorable perception of university POD by those who belong to the STEM area for the reasons already mentioned. Job seniority was also significant in the model, which could be explained by the different reasons that relate the perception of organizational democracy to this variable and which have already been discussed.

Some secondary research results are worth highlighting. The model evidenced the significance of the condition of disability and belonging to indigenous peoples on the POD, the first being the most significant. People with disabilities valued organizational democracy less, which could be reversed if the university improved the policies or strategies that support them. Indeed, the WHO identifies the concept of disabling obstacles to refer to a set of difficulties that a person with disabilities may experience in an organization, such as insufficient policies and standards, insufficient provision of services, negative attitudes, lack of accessibility, and lack of participation. These obstacles can affect the assessment of organizational democracy as long as an inclusive space with equal rights and the absence of prejudice is not observed [104,105]. For its part, traditionally belonging to indigenous peoples has led to expressions of racism in higher education, including in pedagogical practices [106,107]. However, the findings of this study show that this group, which represents 3.8% of the population analyzed, has a positive perception of organizational democracy. This could imply that the public policies of recognition toward this population are perceived positively. Both groups, people with disabilities and those belonging to indigenous peoples, constitute a challenge for organizations in the design of inclusive policies that consider intersectionality with gender [108].

## 5. Conclusions

University professors who are more open to gender equality in roles perceive a greater deployment of organizational democracy, provided they do not perceive discrimination, to which they are particularly sensitive. In this study, we found that the perception of discrimination against women is a good predictor of academics’ views on the perceived level of organizational democracy in the university. The results of our study confirm the perception of discrimination expressed by a high percentage of women. Women also exhibit a greater predisposition toward gender equality. However, it is concerning that academics in STEM fields have a lower inclination toward gender openness. This situation may be explained by the higher presence of men and gender biases in this disciplinary field.

In terms of the perception of organizational democracy, there is a surprising difference between men and women. Women perceive a lower level of organizational democracy, which is linked to their perception of discrimination. In this case, faculty members associated with non-STEM disciplines have a lower appreciation of organizational democracy.

Regarding the perception of discrimination, there is a gap between men and women, with men tending to perceive less discrimination toward women and sexual minorities. It is essential to reduce this gap for the success of gender equality policies.

As for the relationship between the three main variables of this study (POD, PD, and ATGR) and age and job seniority, it is found that young academics are more prone to ATGR than those who are older. In contrast, POD is not related to the age of faculty members but is related to job seniority. This reveals that as one has a greater knowledge of the institution, there is a more skeptical stance toward organizational democracy, which could weaken institutional effectiveness and efficiency.

The perception of discrimination toward women is related to age, with young academics perceiving more discrimination. In the case of the perception of discrimination toward gender and sexual diversity, it is negatively related to age and job seniority.

The practical contribution of this work is to reflect that the success of gender policies depends, to some extent, on reducing the gaps reported in this study. Therefore, universities would do well to reinforce and explain their policies toward gender equality, as it will lead to a better perception of their organizational democracy. In this sense, innovative strategies and relevant support structures are required to impact cultures of discrimination toward women, ensuring the construction of safe and inclusive working environments in higher education. All these issues give possible opportunities for future research since they are relevant in contemporary societies and contribute to understanding social experiences and power relations in academic contexts.

The aforementioned also highlights the need to intervene at the level of organizational culture by mainstreaming the gender perspective in teaching and management in order to promote an ethical change toward respect for diversity and gender equity. This is particularly important given the social role that higher education has in training individuals and their deployment in different spaces of society.

Finally, the results of this study should be considered with some caution regarding the external validity of the conclusions, as the study focuses on only one university, even though the results are in line with previous studies. Internal validity may be affected by voluntary responses. However, the high response rate (58.1%) and post-stratification corrections strengthen the representativeness of the sample and minimize selection bias.

## Figures and Tables

**Table 1:** Items considered in the construction of each index used in this study.

Index	Item (in Terms of the Probability of the Event Occurring)
POD	Fear of speaking out due to possible retaliation. Mistakes are hidden for fear of showing failure. Nothing will change in the University. There are organizational instances open to questioning the decisions of the authorities. Discourse and action are coherent at the University. The University is open to those who propose alternative solutions to the institution’s problems.
PD	PDHM A heterosexual man is recognized for his work. A heterosexual man receives unwanted comments about his appearance. A heterosexual man is affected by discrimination. A heterosexual man is affected by workplace harassment. A heterosexual man is sexually harassed. PDHW A heterosexual woman is recognized for her work. A heterosexual woman receives unwanted comments about her appearance. A heterosexual woman is affected by discrimination. A heterosexual woman is affected by workplace harassment. A heterosexual woman is sexually harassed. PDLGBTQ A person who identifies within the LGBTQ community is recognized for their work. A person who identifies within the LGBTQ community receives unwanted comments about their appearance. A woman who identifies within the LGBTQ community is affected by discrimination. A person who identifies within the LGBTQ community is affected by workplace harassment. A person who identifies within the LGBTQ community is sexually harassed.
ATGR	Women are better teachers. Men are better researchers. Secretaries must be women. There are professional careers for women and men. Being non-heterosexual hinders an academic career. The man is responsible for the family. The woman is responsible for the children and the home. Whoever provides the family income should not do housework. Men are better political leaders. Men’s authority is more secure and stable. Equality is detrimental to women. There are women-only jobs and men-only jobs. Women are better at language and men are better at math. Couples with children should marry. Non-heterosexual people should be able to adopt children.

**Table 2:** Sample characterization.

Variable	Category	Sample Distribution (%) (*n*)	University Distribution (%) (*n*)
Gender	Male	62.4 (255)	69.9 (492)
	Female	37.0 (151)	30.1 (212)
	No response	0.6 (2)	No information ^1^
Nationality	Chilean	95.1 (389)	96.0 (676)
	Other	4.9 (20)	4.0 (28)
Indigenous people	Yes	3.8 (16)	No information
	No	96.2 (393)	No information
Sexual orientation	Heterosexual	93.0 (380)	No information
	Homosexual (gay/lesbian)	3.2 (13)	No information
	Bisexual	1.4 (6)	No information
	No response	2.4 (10)	No information
Age (years)	Low (<40)	17.9 (73)	18.6 (131)
	Medium (≥40; <60)	56.9 (233)	52.2 (367)
	High (≥60)	25.2 (103)	29.2 (206)
Parenthood	Yes	73.9 (302)	No information
	No	26.1 (107)	No information
Contractual modality	Staff/contract	65.5 (268)	62.2 (438)
	Temporary	34.5 (141)	37.8 (266)
Employment seniority (years) ^2^	Low (≤5)	28.1 (115)	No information ^1^
	Medium (>5; ≤15)	30.7 (126)	
	High (>15)	41.2 (169)	
Disciplinary field	STEM	57.3 (234)	49.7 (350)
	Non-STEM	42.7 (175)	50.3 (354)

^1^ It was not possible to access reliable information. ^2^ Employment seniority is the total cumulative number of working years in the university’s academic community.

**Table 3:** Distribution of ATGR, POD, and PD by gender and disciplinary field.

Index ^1^	Variable	Category	Mean Value	SD	Diff ^2^	Distribution (%) ^3^	
						<Cut-Off	≥Cut-Off
ATGR	Gender	Male	3.37	0.41	0.10 **	1.50	98.50
		Female	3.47	0.44		0.69	99.31
	Disciplinary field	STEM	3.34	0.51	0.12 **	1.66	98.34
		Non-STEM	3.46	0.34		0.85	99.15
POD	Gender	Male	2.44	0.53	0.15 **	49.28	50.72
		Female	2.29	0.66		61.31	38.69
	Disciplinary field	STEM	2.48	0.53	0.17 **	48.01	51.99
		Non-STEM	2.32	0.60		57.55	42.45
PDHM	Gender	Male	3.18	0.61	0.38 ***	12.40	87.60
		Female	3.56	0.51		3.48	96.52
	Disciplinary field	STEM	3.34	0.58	0.08	8.30	91.70
		Non-STEM	3.27	0.63		10.25	89.75
PDHW	Gender	Male	2.97	0.70	0.43 ***	21.98	78.02
		Female	2.54	0.87		45.71	54.29
	Disciplinary field	STEM	2.96	0.77	0.26 ***	22.16	77.84
		Non-STEM	2.71	0.75		35.29	64.71
PDLGBTQ	Gender	Male	2.89	0.74	0.30 ***	24.75	75.25
		Female	2.59	0.88		35.55	64.44
	Disciplinary field	STEM	2.90	0.79	0.21 **	23.17	76.83
		Non-STEM	2.69	0.78		32.00	68.00

^1^ ATGR and POD scales: 1–4, 1: very low; 4: very high. PD scale: 1–4, 1: very high; 4: very low. ^2^ *** Significant at the 1 percent level; ** Significant at the 5 percent level; * Significant at the 10 percent level. ^3^ Cut-off value: 2.5.

**Table 4:** Correlation of ATGR, POD, and PD with age and employment seniority.

Index ^1^	Variable	Correlation Value ^2^	Range (Year)	Mean Value ^3^	SD	Significance ^2^
ATGR	Age	−0.1613 ***	Low (<40)	3.53	0.30	Low vs. Medium *
			Medium (≥40; <60)	3.43	0.43	Medium vs. High ***
			High (≥60)	3.26	0.46	Low vs. High ***
	Employment seniority	−0.0559	Low (≤5)	3.38	0.41	Low vs. Medium
			Medium (>5; ≤15)	3.47	0.45	Medium vs. High **
			High (>15)	3.35	0.41	Low vs. High
POD	Age	0.0368	Low (<40)	2.37	0.55	Low vs. Medium
			Medium (≥40; <60)	2.39	0.62	Medium vs. High
			High (≥60)	2.41	0.49	Low vs. High
	Employment seniority	−0.1092 **	Low (≤5)	2.53	0.57	Low vs. Medium **
			Medium (>5; ≤15)	2.34	0.54	Medium vs. High
			High (>15)	2.35	0.58	Low vs. High **
PDHM	Age	−0.0182	Low (<40)	3.43	0.48	Low vs. Medium
			Medium (≥40; <60)	3.26	0.66	Medium vs. High
			High (≥60)	3.31	0.58	Low vs. High
	Employment seniority	−0.0138	Low (≤5)	3.33	0.65	Low vs. Medium
			Medium (>5; ≤15)	3.26	0.60	Medium vs. High
			High (>15)	3.31	0.60	Low vs. High
PDHW	Age	0.1717 ***	Low (<40)	2.61	0.77	Low vs. Medium
			Medium (≥40; <60)	2.81	0.80	Medium vs. High **
			High (≥60)	3.04	0.71	Low vs. High ***
	Employment seniority	0.0490	Low (≤5)	2.80	0.79	Low vs. Medium
			Medium (>5; ≤15)	2.76	0.72	Medium vs. High
			High (>15)	2.91	0.82	Low vs. High
PDLGBTQ	Age	0.1670 ***	Low (<40)	2.56	0.77	Low vs. Medium **
			Medium (≥40; <60)	2.82	0.82	Medium vs. High
			High (≥60)	2.93	0.74	Low vs. High ***
	Employment seniority	0.1103 **	Low (≤5)	2.73	0.79	Low vs. Medium
			Medium (>5; ≤15)	2.70	0.79	Medium vs. High **
			High (>15)	2.92	0.80	Low vs. High *

^1^ ATGR and POD scales: 1–4, 1: very low; 4: very high. PD scale: 1–4, 1: very high; 4: very low. ^2^ *** Significant at the 1 percent level; ** Significant at the 5 percent level; * Significant at the 10 percent level. ^3^ Cut-off value: 2.5.

**Table 5:** Regression model for determining the impact of ATGR, PD, and different control variables on the perception of democracy organizational (POD).

Index or Variable	β non-Standardized Coefficient ^1^	Standard Error	Interval
ATGR	0.4798	0.0602	−0.0701; 0.1661
PDHM	−0.0306	0.0600	−0.1492; 0.0881
PDHW	0.2174 ***	0.0510	0.1169; 0.3180
PDLGBTQ	0.0906 *	0.0501	−0.0088; 0.1900
Gender (female)	0.0204	0.0527	−0.0836; 0.1244
Disciplinary field (STEM)	0.1031 **	0.0469	0.0107; 0.1957
Age	0.0018	0.0022	−0.0026; 0.0062
Employment seniority	−0.0071 **	0.0024	−0.0024; 0.0044
Indigenous people	0.2183 *	0.1170	−0.0053; 0.4418
Disability condition	−0.5192 **	0.2236	−0.9730; −0.0655
Nationality	−0.1419	0.1034	−0.3458; 0.0618
Parenthood	0.1021	0.0587	−0.0134; 0.2177

^1^ *** Significant at the 1 percent level; ** Significant at the 5 percent level; * Significant at the 10 percent level.

## Data Availability

The data presented in this work are available on request from the corresponding author (Elizabeth Troncoso).

## Abbreviations

ATGR	Attitude toward gender roles
LGBTQ	Lesbian, gay, bisexual, transgender, and queer
OD	Organizational democracy
OLS	Ordinary least squares
PD	Perception of gender discrimination
PDHM	Perception of discrimination against heterosexual men
PDHW	Perception of discrimination against heterosexual women
PDLGBTQ	Perception of discrimination against sexual and gender minorities
POD	Perception of organizational democracy
STEM	Science, technology, engineering, and mathematics

## References

[1] Neuhäuser C., Oldenbourg A. (2020). Workplace democracy and corporate human rights responsibilities. Rev. Soc. Econ..

[2] Sosa L. (2021). Beyond gender equality? Anti-gender campaigns and the erosion of human rights and democracy. Neth. Q. Hum. Rights.

[3] de Castro E.S., de Souza I.V., Gomes J.P. (2022). O discurso pós-crítico de gênero e diversidade: Uma propos dos direitos humanos. RIAEE.

[4] Ministerio de Desarrollo Social de Chile (Ministry of Social Development of Chile) (2022). Informe de Desarrollo Social 2022 [Social Development Report 2022]. https://www.desarrollosocialyfamilia.gob.cl/storage/docs/ids/Informe-desarrollo-social-2022.pdf.

[5] Instituto Nacional de Estadísticas de Chile (National Institute of Statistics of Chile) (2023). Estadísticas de Género [Gender Statistics]. https://www.estadisticasdegenero.cl/.

[6] Instituto Nacional de Estadísticas de Chile (National Institute of Statistics of Chile) (2021). Síntesis de Resultados: Encuesta Suplementaria de Ingresos ESI 2021 [Summary of Results: Supplementary Survey of Income ESI 2021]. https://www.ine.gob.cl/docs/default-source/encuesta-suplementaria-de-ingresos/publicaciones-y-anuarios/s%C3%ADntesis-de-resultados/2021/s%C3%ADntesis-nacional-esi-2021.pdf.

[7] Comisión Económica para América Latina y el Caribe (Economic Commission for Latin America and the Caribbean) (2022). Panorama Social de América Latina y el Caribe 2022 [Social Panorama of Latin America and the Caribbean 2022]. https://www.cepal.org/es/publicaciones/48518-panorama-social-america-latina-caribe-2022-la-transformacion-la-educacion-como.

[8] Wodon T., de la Brière B. (2018). Unrealized Potential: The High Cost of Gender Inequality in Earnings. The Cost of Gender Inequality.

[9] Ministerio de Hacienda (Ministry of Finance), Ministerio de Economía, Fomento y Turismo (Ministry of Economy, Development and Tourism), Fundación Chile Mujeres (Chile Women’s Foundation), Organización internacional del Trabajo (International Labor Organization) (2022). Cuarto Reporte de Indicadores de Género en las Empresas en Chile 2022 [Fourth Report on Gender Indicators in Companies in Chile 2022]. https://www.economia.gob.cl/wp-content/uploads/2023/03/original-cuarto-reporte-indicadores-genero-2022-digital.pdf.

[10] Instituto Nacional de Estadísticas de Chile (National Institute of Statistics of Chile) (2017). Censo de Población y Vivienda [Population and Housing Census]. https://www.ine.gob.cl/estadisticas/sociales/censos-de-poblacion-y-vivienda/censo-de-poblacion-y-vivienda.

[11] Ministerio de Desarrollo Social de Chile (Ministry of Social Development of Chile) (2020). Documento de Resultados: Personas Mayores, Envejecimiento y Cuidados [Outcome Document: Elderly People, Aging and Care]. http://observatorio.ministeriodesarrollosocial.gob.cl/storage/docs/grupos-poblacion/Documento_de_resultados_Personas_mayores_envejecimiento_y_cuidados_31.07.2020.pdf.

[12] The World Bank (2022). Indicators: Net Migration—Chile. https://datos.bancomundial.org/indicator/SM.POP.NETM?locations=CL.

[13] United Nations (1948). Universal Declaration of Human Rights. https://www.un.org/en/about-us/universal-declaration-of-human-rights.

[14] United Nations (1993). Declaration on the Elimination of Violence against Women. https://www.ohchr.org/en/instruments-mechanisms/instruments/declaration-elimination-violence-against-women.

[15] United Nations (1995). The Beijing Declaration and Platform for Action. https://www.unwomen.org/en/digital-library/publications/2015/01/beijing-declaration.

[16] Biblioteca del Congreso Nacional de Chile (Library of the National Congress of Chile) Ley N°21.369 Regula el Acoso Sexual, la Violencia y la Discriminación de Género en el Ámbito de la Educación Superior [Law No. 21.369 Regulates Sexual Harassment, Violence and Gender Discrimination in Higher Education]. Diario Oficial de la República de Chile (Official Gazette of the Republic of Chile). 30 August 2021. https://www.bcn.cl/leychile/navegar?idNorma=1165023.

[17] Tokay Ö., Eyupoglu S.Z. (2018). Employee perceptions of organisational democracy and its influence on organisational citizenship behaviour. S. Afr. J. Bus. Manag..

[18] Han K.-S., Garg P. (2018). Workplace democracy and psychological capital: A paradigm shift in workplace. Manag. Res. Rev..

[19] Geçkil T. (2022). Perceived organizational democracy and associated factors: A focused systematic review based on studies in Turkey. Front. Psychol..

[20] Svendsen M., Jønsson T.F. (2022). Organizational democracy and meaningful work: The mediating role of employees corporate social responsibility perceptions. Front. Psychol..

[21] Rezaei M., Ferraris A., Busso D., Rizzato F. (2022). Seeking traces of democracy in the workplace: Effects on knowledge sharing. J. Knowl. Manag..

[22] Adobor H. (2020). Open strategy: Role of organizational democracy. J. Strategy Manag..

[23] National Academies of Sciences, Engineering, and Medicine (2018). Sexual Harassment of Women: Climate, Culture, and Consequences in Academic Sciences, Engineering, and Medicine.

[24] Sexton J., Fairchild E., Newman H., Riggs E., Hinerman K. (2022). University Title IX Requirements have chilling effect on gender discrimination research: A call for a more nuanced approach. J. Interpers. Violence.

[25] Chan D.K.-S., Chow S.Y., Lam C.B., Cheung S.F. (2008). Examining the job-related, psychological, and physical outcomes of workplace sexual harassment: A meta-analytic review. Psychol. Women Q..

[26] Baker S.J., Lucas K. (2017). Is it safe to bring myself to work? Understanding LGBTQ experiences of workplace dignity. Can J. Adm. Sci..

[27] Denier N., Waite S. (2019). Sexual orientation at work: Documenting and understanding wage inequality. Sociol. Compass.

[28] Bagilhole B., White K. (2013). Gender and Generation in Academia.

[29] Chaves Jiménez R. (2015). Aspectos relevantes para la transversalización de la perspectiva de género en el proceso de desarrollo curricular universitario. Rev. Espiga.

[30] Ellemers N. (2018). Gender stereotypes. Annu. Rev. Psychol..

[31] Taylor E.A., Smith A.B., Welch N.M., Hardin R. (2018). You should be flattered!: Female sport management faculty experiences of sexual harassment and sexism. Women Sport. Phys. Act. J..

[32] O’Connor K.W., Drouin M., Niedermeyer T. (2021). How do age, sex, political orientation, religiosity, and sexism affect perceptions of sex assault/harassment allegations?. Sex Cult..

[33] Kirkner A.C., Lorenz K., Mazar L. (2022). Faculty and staff reporting & disclosure of sexual harassment in higher education. Gend. Educ..

[34] André S., Gesthuizen M., Scheepers P. (2013). Support for traditional female roles across 32 countries: Female labour market participation, policy models and gender differences. Comp. Sociol..

[35] Merma-Molina G., Ávalos Ramos M., Martínez Ruiz M. (2017). La igualdad de género en la docencia universitaria. Percepciones del alumnado. Manzana Discordia.

[36] Thijs P., Te Grotenhuis M., Scheepers P. (2017). The relationship between societal change and rising support for gender egalitarianism among men and women: Results from counterfactual analyses in The Netherlands, 1979–2012. Soc. Sci. Res..

[37] Bondestam F., Lundqvist M. (2020). Sexual harassment in higher education—A systematic review. Eur. J. High. Educ..

[38] Henning M.A., Zhou C., Adams P., Moir F., Hobson J., Hallett C., Webster C.S. (2017). Workplace harassment among staff in higher education: A systematic review. Asia Pac. Educ. Rev..

[39] McKay R., Arnold D.H., Fratzl J., Thomas R. (2008). Workplace bullying in academia: A Canadian study. Empl. Responsib. Rights J..

[40] Geçkil T., Tikici M. (2015). A study on developing the organizational democracy scale. Amme Idaresi Derg..

[41] Ahmed K., Adeel A., Ali R., Rehman R.U. (2019). Organizational democracy and employee outcomes: The mediating role of organizational justice. Bus. Strategy Dev..

[42] Ahmed K., Ahmed A. (2022). The rationale and development of organizational democracy scale. Bus. Politics.

[43] Çopur Z., Atanur Baskan G. (2020). Örgütsel Demokrasi ile Örgütsel Sinizm Arasındaki İlişki: Öğretim Elemanları Üzerine Bir Araştırma. Yükseköğretim Derg..

[44] Turabik T., Atanur Baskan G. (2020). The relationship between organizational democracy and political behaviors in universities. J. Appl. Res. High. Educ..

[45] UNESCO (2014). UNESCO Priority Gender Equality Action Plan: 2014–2021.

[46] Acar-Erdol T., Bostancioglu A., Gözütok F.D. (2022). Gender equality perceptions of preservice teachers: Are they ready to teach it?. Soc. Psychol. Educ..

[47] O’Connor P., Irvine G. (2020). Multi-level state interventions and gender equality in higher education institutions: The Irish case. Adm. Sci..

[48] Araya Umaña S. Políticas de igualdad de género y educación superior: Desafíos conceptuales y prácticos. Proceedings of the International Seminar Quality of Higher Education and Gender.

[49] Zambrano C., Perugache A., Figueroa J. (2017). Manifestaciones de la violencia basada en género en docentes universitarios. Psicogente.

[50] Pérez A., Betancourt M. (2019). El enfoque de género desde la formación docente y su relación con la ciencia, la tecnología y la sociedad. Rev. Boletín Redipe.

[51] Zambrano C., Matabanchoy S.M. (2022). Relación entre calidad de vida en el trabajo y roles de género en docentes universitarios. Rev. Boletín Redipe.

[52] Hernández D.E., Jiménez V.M., Ballesteros C.A. (2019). La comunidad universitaria, frente a la igualdad de género: Un estudio cuantitativo. Rev. Lat. Comun. Soc..

[53] Pla-Julián I., Díez J.-L. (2019). Gender equality perceptions of future engineers. Eng. Stud..

[54] Kitta I., Cardona-Moltó M.-C. (2022). Students’ perceptions of gender mainstreaming implementation in university teaching in Greece. J. Gend. Stud..

[55] Llorent-Bedmar V., Llorent-Vaquero M., Navarro-Granados M. (2017). Towards gender equality in Moroccan universities: Female university teachers from a gender perspective. Women’s Stud. Int. Forum.

[56] Blättel-Mink B., Kuhlmann E. (2003). Health professions, gender and society: Introduction and outlook. Int. J. Sociol. Soc. Policy.

[57] Montané A., Pessoa de Carvalho M.E. (2012). Diálogo sobre género: Justicia, equidad y políticas de igualdad en educación superior (Brasil y España). Rev. Lusófona Educ..

[58] Evetts J. (2014). Women and Career: Themes and Issues in Advanced Industrial Societies.

[59] Casad B.J., Garasky C.E., Jancetic T.R., Brown A.K., Franks J.E., Bach C.R. (2022). U.S. women faculty in the Social Sciences also face gender inequalities. Front. Psychol..

[60] McCarry M., Jones C. (2022). The equality paradox: Sexual harassment and gender inequality in a UK university. J. Gend. Stud..

[61] Bagilhole B. (2002). Academia and the reproduction of unequal opportunities for women. Sci. Technol. Stud..

[62] Settles I.H., Cortina L.M., Malley J., Stewart A.J. (2006). The climate for women in academic science: The good, the bad, and the changeable. Psychol. Women Q..

[63] Casad B.J., Franks J.E., Garasky C.E., Kittleman M.M., Roesler A.C., Hall D.Y., Petzel Z.W. (2021). Gender inequality in academia: Problems and solutions for women faculty in STEM. J. Neurosci. Res..

[64] Domínguez A., Diez R. (2022). Gender barriers in academia: Perceptions of inequality in professional development among female academics in the Faculty of Education, University of Alicante, Spain. Societies.

[65] Buquet A., Cooper J.A., Mingo A., Moreno H. (2013). Intrusas en la Universidad.

[66] Lee K.S., Tufiş P.A., Alwin D.F. (2010). Separate spheres or increasing equality? Changing gender beliefs in postwar Japan. J. Marriage Fam..

[67] Constantin A., Voicu M. (2015). Attitudes towards gender roles in cross-cultural surveys: Content validity and cross-cultural measurement invariance. Soc. Indic. Res..

[68] Valliant R., Dever J.A. (2018). Survey Weights: A Step-By-Step Guide to Calculation.

[69] CUECH Consorcio de Universidades del Estado de Chile (Council of State Universities of Chile) Tendencias de brechas en las 18 Universidades del CUECH Resumen Ejecutivo [Gap Trends in the 18 CUECH Universities Executive Summary]. https://ethos.uestatales.cl/2022/08/26/tendencias-de-brechas-en-las-18-universidades-del-cuech/.

[70] Eslen-Ziya H., Yildirim T.M. (2022). Perceptions of gendered-challenges in academia: How women academics see gender hierarchies as barriers to achievement. Gend. Work Organ..

[71] Knight C.R., Brinton M.C. (2017). One egalitarianism or several? Two decades of gender-role attitude change in Europe. Am. J. Sociol..

[72] Lomazzi V., Crespi I. (2019). Gender Mainstreaming and Gender Equality in Europe: Policies, Culture and Public Opinion.

[73] Linstead S., Brewis J., Linstead A. (2005). Gender in change: Gendering change. J. Organ. Chang. Manag..

[74] Ramdhany M.A., Komariah A., Hufad A., Kurniady D.A. Competitive advantage and organizational effectiveness at public universities of educational institution of education personnel. Proceedings of the 2nd International Conference on Research of Educational Administration and Management.

[75] Blackmore P. (2007). Disciplinary difference in academic leadership and management and its development: A significant factor?. Res. Post-Compuls. Educ..

[76] Lee J.J., Rhoads R.A. (2004). Faculty entrepreneurialism and the challenge to undergraduate education at research universities. Res. High. Educ..

[77] Kekäle J. (1999). ‘Preferred’ patterns of academic leadership in different disciplinary (sub)cultures. High. Educ..

[78] Marcaletti F., Íñiguez-Berrozpe T., Elboj-Saso C., Garavaglia E. (2022). Adult training as a quality factor in work trajectory: Positive effects of adult training on seniority and ageing at work. Adult Educ. Q..

[79] MacLeod K. (1987). The Seniority Principle: Is it Discriminatory?.

[80] Clarke J.S., Ellett C.D., Bateman J.M., Rugutt J.K. Faculty receptivity/resistance to change, personal and organizational efficacy, decision deprivation and effectiveness in research I universities. Proceedings of the Annual Meeting of the Association for the Study of Higher Education.

[81] Florah O.M. (2019). Age and effective human resource management. Eur. Sci. J..

[82] Gullette M.M. (2018). The monument and the wrecking crew. Academe.

[83] Bird S.R. (2011). Unsettling universities’ incongruous, gendered, bureaucratic structures: A case-study approach. Gend. Work Organ..

[84] Ecklund E.H., Lincoln A.E., Tansey C. (2012). Gender segregation in elite academic science. Gend. Soc..

[85] Rhoads R.A., Gu D.Y. (2012). A gendered point of view on the challenges of women academics in the People’s Republic of China. High. Educ..

[86] Sattari N., Sandefur R.L. (2019). Gender in academic STEM: A focus on men faculty. Gend. Work Organ..

[87] Carr P.L., Ash A.S., Friedman R.H., Szalacha L., Barnett R.C., Palepu A., Moskowitz M.M. (2000). Faculty perceptions of gender discrimination and sexual harassment in academic medicine. Ann. Intern. Med..

[88] Hill K.Q., Hurley P.A. (2022). Perceptions of academic departmental climate by men and women and the effects of such perceptions on research productivity. PS Polit. Sci. Polit..

[89] Gururaj G.P., Manohar J.S., Rao T.S.S. (2023). Attitudes and opinions of the teaching faculty toward the LGBT community. J. Psychosexual Health.

[90] Taracuk M.D., Koch J.M. (2023). Use of a media intervention to increase positive attitudes toward transgender and gender diverse individuals. Int. J. Transgend Health.

[91] Funk C., Parker K. (2018). Women and Men in STEM often at Odds Over Workplace Equity.

[92] Lee Y.J., Na J., Kim B.K. (2022). Age, gender and one’s perception of discrimination against men versus women in Korea. Psychol. Rep..

[93] García-González J., Forcén P., Jimenez-Sanchez M. (2019). Men and women differ in their perception of gender bias in research institutions. PLoS ONE.

[94] Li D., Koedel C. (2017). Representation and salary gaps by race-ethnicity and gender at selective public universities. Educ. Res..

[95] Kehn A., Ruthig J.C. (2013). Perceptions of gender discrimination across six decades: The moderating roles of gender and age. Sex Roles.

[96] Mihajlović Trbovc J., Černič Istenič M., Petrović T., Andreou A. (2022). Structural positions, hierarchies, and perceptions of gender equality: Insights from a Slovenian research organisation. Družboslovne Razpr./Soc. Sci. Forum.

[97] Ordorika I. (2015). Equidad de género en la Educación Superior. Rev. Educ. Super..

[98] Ríos N., Mandiola M., Varas A. (2017). Haciendo género, haciendo academia: Un análisis feminista de la organización del trabajo académico en Chile. Psicoperspectivas.

[99] Alwazzan L., Al-Angari S. (2020). Women’s leadership in academic medicine: A systematic review of extent, condition and interventions. BMJ Open.

[100] Savigny H. (2014). Women, know your limits: Cultural sexism in academia. Gend. Educ..

[101] Howe-Walsh L., Turnbull S. (2016). Barriers to women leaders in academia: Tales from science and technology. Stud. High Educ..

[102] Holman L., Stuart-Fox D., Hauser C.E. (2018). The gender gap in science: How long until women are equally represented?. PLoS Biol..

[103] Thomson A., Palmén R., Reidl S., Barnard S., Beranek S., Dainty A.R.J., Hassan M.T. (2022). Fostering collaborative approaches to gender equality interventions in higher education and research: The case of transnational and multi-institutional communities of practice. J. Gend. Stud..

[104] Kabia E., Mbau R., Muraya K.W., Morgan R., Molyneux S., Barasa E. (2018). How do gender and disability influence the ability of the poor to benefit from pro-poor health financing policies in Kenya? An intersectional analysis. Int. J. Equity Health.

[105] Rohmer O., Louvet E. (2018). Implicit stereotyping against people with disability. Group Process Intergroup Relat..

[106] Tate S.A., Bagguley P. (2017). Building the anti-racist university: Next steps. Race Ethn. Educ..

[107] Brahimi M.A., Idir M. (2020). Études postcoloniales et sciences sociales: Pistes d’analyse pour un croisement théorique et épistémologique. Rev. Interv. Écon..

[108] Lombardo E., Rolandsen L. (2012). Framing gender intersections in the European Union: What implications for the quality of intersectionality in policies?. Soc. Polit. Int. Stud. Gend. State Soc..

